# Pharmacist-Prescribed Hormonal Contraception: Does Didactic Hormonal Contraception Education Affect Student Pharmacist Perceptions of This Professional Activity?

**DOI:** 10.3390/pharmacy9030145

**Published:** 2021-08-20

**Authors:** Rachel Rikard, Jennifer Elliott, Erin Dalton, Rebecca H. Stone

**Affiliations:** 1 College of Pharmacy, University of Georgia, Athens, GA 30602, USA; rachelarikard@gmail.com; 2 College of Pharmacy, Mercer University, Atlanta, GA 30041, USA; JSaddana@ITS.JNJ.com; 3 School of Pharmacy, South University, Savannah, GA 31406, USA; eedalton@southuniversity.edu

**Keywords:** hormonal contraception, student pharmacists, pharmacy access, pharmacist-prescribed

## Abstract

Since 2014, select states have allowed pharmacists to prescribe hormonal contraception (HC). This study describes student pharmacists’ perceptions of a pharmacist’s scope of practice, education, and interest, and identifies differences between students who have completed didactic HC content in their professional curriculum versus those who have not. A voluntary online survey was emailed to all students in three Georgia pharmacy schools. Descriptive statistics were reported. Likert square responses were dichotomized, and Chi square testing identified differences between groups. A total of 1256 students were invited, 35% completed the survey, of those 68% had received HC didactic content in their curriculum. Regardless of HC education, most students “agree” or “strongly agree” that pharmacists are adequately educated to prescribe HC (92% vs. 86%, *p* = 0.05) and prescribing HC is within the pharmacist’s scope of practice (89% vs. 84%, *p* = 0.12). Although not currently permitted in Georgia, most are interested in prescribing (97% vs. 96%, *p* = 0.5). Of the students who have received HC didactic content, 87% felt “moderately”, “well”, or “extremely well-educated” regarding HC prescribing clinical skills. Regardless of didactic training, pharmacy students believe pharmacists are prepared to prescribe HC and support pharmacist-prescribed HC as a part of their future professional scope of practice.

## 1. Introduction

In 2011, 45% of pregnancies in the United States were unintended [1]. Pharmacist-prescribed hormonal contraception (HC) is one strategy that has been implemented in an attempt to improve patient access and uptake of these medications. Multiple studies, including a systematic review of these data, have assessed patient, pharmacist, and pharmacy student attitudes and interest in pharmacist-prescribed HC. Data indicate that a majority in all three groups support this expanded scope of practice, and intend to participate if available in their state [2,3,4,5,6].

In 2016, California and Oregon successfully implemented pharmacist-prescribed contraception without a physician’s prescription, and over the past five years this practice has continued to steadily expand [7,8]. Currently, 16 U.S. states or jurisdictions allow pharmacists to prescribe hormonal, and in some states emergency contraceptives, under statewide statutes or regulations, with more underway [7,8]. As public and professional acceptance of pharmacist-prescribed contraception has grown, it is reasonable to believe that pharmacy student training experiences and expectations of their professional activities may have also changed compared to pharmacists who started practicing more than 5 years ago. Georgia does not currently permit pharmacist-prescribed HC, but recent data from other non-prescribing states indicate that student pharmacists in their last year of their didactic training (third professional year) currently view this as a professional responsibility [9]. 

This study aims to assess pharmacy student’s (1) perceptions of pharmacist education, (2) perceptions of a pharmacist’s scope of practice regarding contraceptive prescribing, and (3) personal interest in providing this service as a pharmacist, and compare differences in these variables between students who have received contraceptive education versus those who have not. Secondary aims include describing the perceptions of students who have received contraceptive education regarding their comfort with prescribing specific contraceptive products and perceived benefits and barriers to prescribing HC. 

## 2. Materials and Methods

This study was determined to be exempt by the University of Georgia’s Review Board. The voluntary survey tool was developed based on previously published research and included Likert-type and multiple-choice questions.

Students from three pharmacy schools in the state of Georgia (University of Georgia, Mercer University, and South University) were invited to participate in a survey via email. The initial invitation was sent at the end of the fall semester/quarter in late November 2020, followed by two reminder emails, and the survey closed at the beginning of the spring semester/quarter, the first week of February, 2021. Students were offered the opportunity to enter a raffle for one of three $50 Amazon gift cards upon completion of the survey. The survey took approximately 10 min to complete and was administered entirely online.

Timing of didactic HC content in the pharmacy curricula of each school was used to categorize students into those who have or have not yet received HC education. Students covered didactic HC content in the fall semester of P3 year at Mercer University, fall semester of P2 year at University of Georgia, and the last quarter of P2 year at South University. Didactic HC content was defined as the primary classroom-based activity used to teach this topic area, and place in the curriculum was identified through email correspondence with the faculty members that teach this content at each school of pharmacy.

The survey consisted of multiple-choice and free text questions assessing demographic information, pharmacy work experience, and interest in prescribing HC if permitted in Georgia. The second portion of the survey consisted of 5-point Likert-type questions assessing student’s comfort level, knowledge, and perceptions of pharmacist-prescribed HC. The full survey is available in Appendix A and Appendix B.

Descriptive statistics were used and reported as percentages based on survey results. Likert square responses were dichotomized, and Chi square testing identified differences between groups.

## 3. Results

Of the 1256 students invited, 35% (*n* = 436) participated in the survey and were included in data analysis. Table 1 contains demographic information for all participants. Average participant age was 25 years old (standard deviation = 3.6) and the majority (73%) were female. Although students from one institution comprised just over half the students included in the survey, the response rate based on school enrollment size was more comparable (University of Georgia 42%, Mercer University 30%, and South University 27%). Participants across the professional curriculum were included, with around 25% of students responding from each class.

Nearly 90% of student pharmacists reported having pharmacy work experience. Table 2 represents student work experience stratified by year in school. Most participants (84%) reported community pharmacy work experience in either a chain or independent setting. Most participants reported greater than a year of community pharmacy work experience, with either one to two years (27%) or three to four years (25%), respectively. When asked if students planned to work in a community pharmacy upon graduation, over one-fourth (28%) of participants responded “yes”, 40% were “unsure”, and 33% responded that they do not plan to work in a community pharmacy upon graduating.

Of the 436 students that completed the survey, 68% had previously received didactic HC education in their professional curriculum and 32% had not. Although a larger percentage of students that received didactic education “agreed” or “strongly agreed” that pharmacists are adequately educated to prescribe HC (92% vs. 86%, *p* = 0.05), the rate of agreement was high within both groups. Further, a similar rate of students in both groups “agreed” or “strongly” agreed that prescribing HC is within the pharmacist’s scope of practice (89% vs. 84%, *p* = 0.12). The vast majority of students in both groups reported that they would be interested in prescribing HC if it was permitted in Georgia (97% vs. 96%, *p* = 0.51).

**Figure 1:**
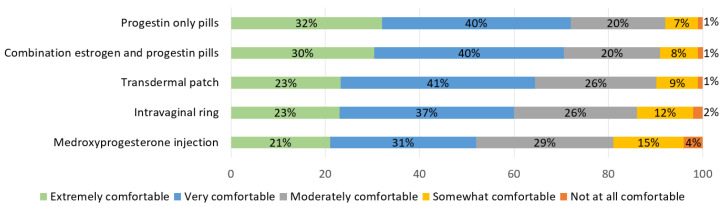
Pharmacy student comfort level prescribing hormonal contraception products after didactic content (*n* = 296).

Of the 296 students who had previously received HC education, 62% reported that their pharmacy curricula left them “moderately educated”, “well educated”, or “extremely well educated” to provide the clinical skills needed to for pharmacist-prescribed HC. However, 35% of students were “not at all familiar” or “somewhat familiar” with the US Centers for Disease Control and Prevention: Medical Eligibility Criteria for Contraceptive Use (USMEC). Figure 1 depicts the student-reported knowledge and comfort level with prescribing specific methods of HC. Students were most comfortable with oral contraceptive pills, with 71% and 72% reporting that they were “very comfortable” or “extremely comfortable” with combination estrogen and progestin and progestin only pills, respectively. Students were least comfortable with prescribing medroxyprogesterone injection, with 52% of students reporting that they were “very comfortable” or “extremely comfortable” with this method.

**Figure 2:**
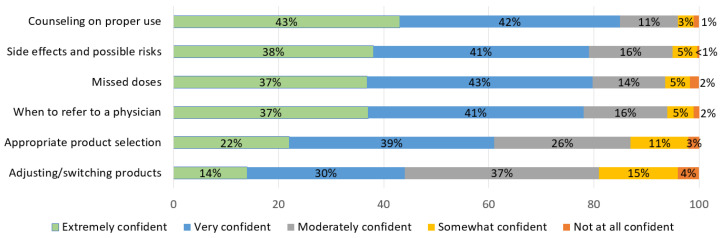
Pharmacy student confidence level in providing hormonal contraception services after didactic content (*n* = 296).

Student pharmacists’ confidence level in providing services related to prescribing HC is shown in Figure 2. Pharmacy students who received HC education were most confident with counseling on proper use of HC, with 85% reporting they felt “very confident” or “extremely confident”. However, only 44% of students reported being “very confident” or “extremely confident” regarding adjusting and/or switching HC products.

**Figure 3:**
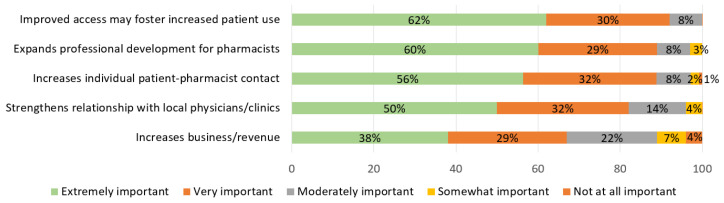
Pharmacy student perceived benefits of prescribing hormonal contraception after didactic content (*n* = 296).

Pharmacy students ranked the importance of perceived benefits of pharmacist-prescribed HC. See Figure 3. Participants most often reported it is “very important” or “extremely important” to improve access that may foster increased patient use (91%), expand professional development for pharmacists (89%), and increase individual patient–pharmacist contact (89%). Further, 10.8% of students reported that increasing business/revenue was “somewhat important” or “not important”.

**Figure 4:**
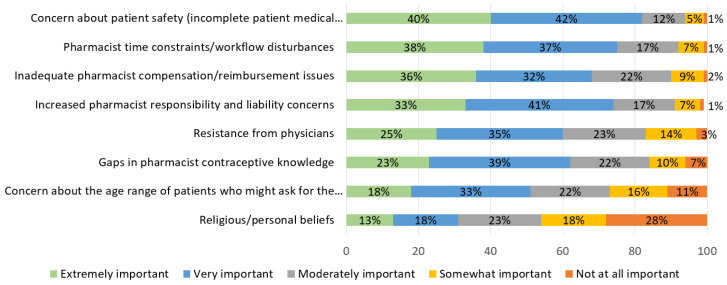
Pharmacy student perceived barriers of prescribing hormonal contraception after didactic content (*n* =296).

Participants were also asked to rank the importance of perceived barriers to pharmacist-prescribed HC. See Figure 4. Perceived barriers most commonly ranked either “very” or “extremely important” were concern about patient safety (82%), pharmacist time constraints/workflow disturbances (75%), and increased pharmacist responsibility and liability concerns (75%). Almost half of students (46%) reported that religious/personal beliefs were somewhat important or not important at all.

## 4. Discussion

The results of this study demonstrate that regardless of timing within their professional training, current student pharmacists (1) feel pharmacists are adequately educated to prescribe HC, (2) embrace the expansion of the pharmacist’s scope of practice to include prescribing HC, and (3) are interested in prescribing HC as part of their future careers. Regardless of training status, well over 80% of students “agreed” or “strongly agreed” with each of these aforementioned statements, which is comparable to other student reported perspectives [4,9].

A 2007 survey assessing California pharmacy students in their second or third professional years who had completed contraceptive training identified very strong interest in pharmacist prescribing (96.2%) [4]. Additionally, Mospan et al. found that 83.6% of third year pharmacy students believe prescribing contraception is a professional responsibility [9]. However, survey data from practicing pharmacists indicate that although a majority are interested in prescribing contraception, the rates are generally lower than those reported by students. National surveys in 2009 and 2019 found that 85% and 65% of pharmacists were interested in prescribing contraception [3,5]. Following the approval of legislation permitting pharmacist-prescribed HC, but before it was actually implemented, studies found that 57% of Oregon pharmacists reported that they would potentially be interested in prescribing contraception if adequate training and reimbursement were offered [10], and 72.7% of pharmacists in California reported that they planned to implement this service [11].

Even more notable, the actual implementation of pharmacist prescribing has varied across the states where it is permitted, and is lower than expected based on surveys of pharmacist interest. Early studies conducted in California found only about 10% of pharmacies offered this service [12,13]. Rodriguez et al. determined that only 63% of Oregon zip codes had at least one pharmacist able to prescribe contraception one year after the state implemented policies [14], and in 2019 only 42% of pharmacies in Oregon and New Mexico offered HC prescribing [15].

The fact that pharmacy student interest in prescribing HC is higher than pharmacist reported interest and actual implementation likely stems from multiple factors. While the majority of pharmacists support pharmacist-prescribed HC and express an interest in providing contraceptive services, they also commonly report concerns regarding lack of compensation and time constraints [3,5]. The transition from student to pharmacist typically includes increased professional responsibilities and related time constraints [16,17], which could plausibly decrease individual enthusiasm to pursue this activity. Although not directly reported in the literature, concerns regarding lack of compensation and time constraints are also likely held by pharmacy retail corporations and pharmacy owners. Pharmacists who are interested in prescribing HC, particularly those in chain organizations, are likely unable to do so without the support of their employer. Support from pharmacy retail corporations, employers, and insurance providers is needed to develop effective solutions for reimbursement/payment and time constraint concerns.

This study found that a high percentage of current pharmacy students felt adequately prepared to prescribe HC. The previously mentioned 2007 survey conducted by Rafie et al. found that 65% of students who had received HC content in their curriculum personally felt adequately educated to prescribe HC [4], compared to 87% in this study who felt “moderately”, “well”, or “extremely well” educated to prescribe hormonal contraception. In addition to slightly different wording, this difference may be influenced by the fact that HC prescribing was embraced in pharmacy practice before most students in this study became interested/introduced to the profession of pharmacy; therefore, most have been introduced to this activity as an established component of the pharmacist’s expanding role.

Additionally, pharmacy student confidence in their ability to prescribe HC may be related to changes in their training. One national survey of practicing pharmacists, of which >40% of participants had been in practice over 20 years, indicated that although a majority felt they had received adequate training to prescribe HC, 27% reported it was not covered in their pharmacy school curriculum [18]. As pharmacist-prescribed HC has been integrated into practice, many schools of pharmacy have strengthened this area of the curriculum, even in states that do not currently allow this practice [19,20]. Rim et al. conducted a recent survey assessing the contraceptive curricula taught in pharmacy programs nationwide and found that 68% of programs reported feeling their HC education was adequate, but 70% also expressed an interest in access to a standardized HC curricula [21]. The successful implementation of pharmacist-prescribed HC, including in states that allow new graduates to prescribe HC without additional training outside of the PharmD curriculum, demonstrates that modern PharmD curricula are adequate in preparing students for this task [22]. However, when evaluating student preparation for this activity, there is sparse data evaluating different teaching methodologies or competency outcomes [22]. Additional research is needed to identify highly effective teaching methods that prepare students for this activity, ideally reporting data that do not rely exclusively on subject self-perception.

As expected, students in this study were most confident with product counseling and side effects, likely in part because this has traditionally been one of the mainstays of pharmacist training for all medication types. Students in this study and other studies exhibited less confidence in switching between HC products and choosing appropriate product selection [4,20]. Schools of pharmacy may need to continue to adapt HC curriculum content and provide additional training, particularly for clinical activities such as selecting and switching between products, to increase the percentage of students who feel confident in these areas.

In Georgia, student perceived benefits and barriers were similar to previous studies conducted with pharmacists and pharmacy students in other states [3,4,5,6]. The top three benefits that students identified as important were (1) improving access to foster increased patient use, (2) expanding professional development, and (3) increasing patient contact. The top three barriers were (1) concerns about patient safety, (2) pharmacist time constraints, and (3) payment/reimbursement issues.

Similar to all studies, there were limitations to this research. The results of this voluntary survey may have been subject to selection bias. One college of pharmacy in Georgia did not participate, and the survey was limited to a single state. Two colleges of pharmacy were four year programs with a semester schedule, and one was a three year program with a quarter schedule. Didactic hormonal contraception content differed in timing within each curriculum, was delivered by different faculty members, and was not standardized across schools. Additionally, differences in other educational and professional experiences could not be controlled for and likely also differ across schools.

## 5. Conclusions

This study found that in a state without pharmacist prescribing, the vast majority of student pharmacists, regardless of professional year or education received, believe that (1) pharmacists are adequately educated to prescribe HC, (2) prescribing HC is part of pharmacists’ professional scope of practice, and (3) are interested in prescribing HC as part of their future careers. This highlights a significant change in perceptions of the pharmacist scope of practice in the United States, where modern students now see prescribing as part of their future career, regardless of whether or not the authority is currently available in their state.

Most pharmacy students believe their curriculum has adequately prepared them for HC prescribing, although additional research is needed to identify what types of training are needed to improve confidence with complex clinical activities, such as switching between products, and referral for alternatives such as IUDs, surgical procedures, or other non-hormonal products.

## Figures and Tables

**Table 1:** Pharmacy student demographics (*n* = 436).

Characteristic	% (*n*)
Male	27.5 (120)
Female	72.5 (316)
Doctor of Pharmacy Program	
Mercer University	32.3 (141)
South University	14.2 (62)
University of Georgia	53.4 (233)
Year in Pharmacy School	
P1—First year	25 (109)
P2—Second year	22.7 (99)
P3—Third year	25.5 (111)
P4—Fourth year	26.8 (117)
Students who have received HC didactic content	67.9 (296)
Students who have not received HC didactic content	32.1 (140)

**Table 2:** Pharmacy student work experiences (%).

Year in School	Community—Independent	Community—Chain	Hospital	Other	No Work Experience
P1	14.7	65.1	11.9	2.8	20.2
P2	30.3	64.6	14.1	3	14.1
P3	19.8	76.6	35.1	7.2	4.5
P4	36.8	72.6	41.9	11.1	4.3
Total	25.5	70	26.4	6.2	10.6

## Data Availability

The data presented in this study are available on request from the corresponding author. The data are not publicly available due to subject privacy.

## Appendix A

University of Georgia Consent Letter

Pharmacy Student Survey: Opinions of Pharmacist-Prescribed Hormonal Contraception

Dear Participant,

My name is Rebecca Stone and I am a faculty member at the University of Georgia College of Pharmacy. I am inviting you to take part in a research study. Pharmacists are able to prescribe hormonal contraception in twelve states. I am conducting research to document the opinions and perceptions of pharmacy students in Georgia regarding pharmacist- prescribed hormonal contraception.

I am surveying students from all accredited Doctorate of Pharmacy programs in Georgia. If you agree to take part in this study, you will be asked to complete a 10 minute survey. Your responses may help us understand how pharmacist prescribed contraception is perceived by early career pharmacists in Georgia.

Participation in this survey is voluntary. You can refuse to take part or stop at any time without penalty, and your decision to participate will have no impact in your grades or coursework. You may skip questions if you do not wish to answer them. Your responses to the survey will be anonymous. The data will be analyzed in aggregate, and reported with descriptive statistics. This research involves the transmission of data over the Internet. Every reasonable effort has been taken to ensure the effective use of available technology; however, confidentiality during online communication cannot be guaranteed.

For your participation, you will be entered into a drawing for one of three $50 gift cards to Amazon. You do not have to be in the study to enter the drawing. You may enter the drawing through a link at the end of the survey, or you may send an email to rhstone@uga.edu to enter the drawing if you do not want to be in the study. Your name will be provided to the investigator’s departmental business office for tracking purposes if you win.

If you have questions about this research, please feel free to contact me at rhstone@uga.edu. If you have any complaints or questions about your rights as a research volunteer, contact the IRB at 706-542-3199 or by email at IRB@uga.edu. Please keep this letter for your records.

Sincerely,

Rebecca Stone, PharmD—University of Georgia College of Pharmacy

Jennifer B. Elliott, PharmD—Mercer University College of Pharmacy

Erin E. Dalton, PharmD—South University School of Pharmacy

## Appendix B

Survey Instrument

Pharmacy Student Survey: Opinions of Pharmacist-Prescribed Hormonal Contraception

**Demographic Information**

- Gender
  □ Male
  □ Female
- Age: _________
- College of Pharmacy
  □ University of Georgia
  □ Mercer University
  □ South University
  □ Philadelphia College of Osteopathic Medicine
- Pharmacy School Year
  □ P1
  □ P2
  □ P3
  □ P4

**Pharmacy Experience**

- What type of pharmacy work experience do you have?
  □ Community
    - What was the setting of your community pharmacy work experience?
      □ Chain pharmacy
      □ Independent pharmacy
      □ Other: __________
    - How long have you worked for a community pharmacy?
      □ Less than 1 year
      □ 1–2 years
      □ 3–4 years
      □ 5 years+
  □ Hospital
  □ Other: _________
  □ No pharmacy work experience
- Do you plan to work in a community pharmacy upon graduation?
  □ Yes
  □ No
  □ Unsure at this time

**What is your attitude towards the following general statements?**

- Prescribing hormonal contraceptives is within the pharmacist’s scope of practice
  □ Strongly disagree
  □ Disagree
  □ Neutral
  □ Agree
  □ Strongly agree
- Pharmacists are adequately educated to prescribe and counsel for hormonal contraception
  □ Strongly disagree
  □ Disagree
  □ Neutral
  □ Agree
  □ Strongly agree
- If it was permitted in Georgia, would you be interested in prescribing hormonal contraception?
  □ Yes, for women 18 and older
  □ Yes, for women 17 and older (legal age for marriage)
  □ Yes, for women 13 and older
  □ No, I am not interested in providing this service
    - Can you share specific reasons why you would opt out of providing pharmacist-prescribed contraception? ________________
- How do you feel that your pharmacy curricula thus far has provided education of clinical skills related to pharmacist-prescribed hormonal contraception?
  □ Do not feel educated
  □ Feel somewhat educated
  □ Feel moderately educated
  □ Feel well educated
  □ Feel extremely well educated
- How familiar are you with the US Centers for Disease Control and Prevention: Medical Eligibility Criteria for Contraceptive Use (USMEC)?
  □ Not at all familiar
  □ Somewhat familiar
  □ Moderately familiar
  □ Very familiar
  □ Extremely familiar

**If allowed in your state, how comfortable would you be prescribing the following methods of contraception?**

	**Not at All Comfortable**	**Somewhat Comfortable**	**Moderately Comfortable**	**Very Comfortable**	**Extremely Comfortable**
Combination estrogen and progestin pills					
Progestin-only pills					
Transdermal patch					
Intravaginal ring					
Medroxyprogesterone injection (Depo-Provera)					

**What is your confidence level in providing the following hormonal contraception services:**

	**Not at All Confident**	**Somewhat Confident**	**Moderately Confident**	**Very Confident**	**Extremely Confident**
Appropriate product selection					
Adjusting/switching products					
Counseling on proper use					
Missed doses					
When to refer to a physician					
Side effects and possible risks					

**Rate the importance of benefits related to pharmacist-prescribed hormonal contraception:**

	**Not at All Important**	**Somewhat Important**	**Moderately Important**	**Very Important**	**Extremely Important**
Improved access may foster increased patient use					
Expands professional development for pharmacists					
Increases individual patient–pharmacist contact					
Strengthens relationship with local physicians/clinics					
Increases business/revenue					

**Rate the importance of barriers related to pharmacist-prescribed hormonal contraception:**

	**Not at All Important**	**Somewhat Important**	**Moderately Important**	**Very Important**	**Extremely Important**
Pharmacist time constraints/workflow disturbances					
Concern about patient safety (incomplete patient medical record, women neglecting annual exam)					
Increased pharmacist responsibility and liability concerns					
Inadequate pharmacist compensation/reimbursement issues					
Gaps in pharmacist contraceptive knowledge					
Resistance from physicians					
Concern about the age range of patients who might ask for the service					
Religious/personal beliefs					
